# Dietary long-chain, but not medium-chain, triglycerides impair exercise performance and uncouple cardiac mitochondria in rats

**DOI:** 10.1186/1743-7075-8-55

**Published:** 2011-08-01

**Authors:** Andrew J Murray, Nicholas S Knight, Sarah E Little, Lowri E Cochlin, Mary Clements, Kieran Clarke

**Affiliations:** 1 Department of Physiology, Anatomy & Genetics, University of Oxford, Parks Rd, Oxford, OX1 3PT, UK

**Keywords:** Exercise, metabolism, mitochondria, fatty acids

## Abstract

Short-term consumption of a high-fat diet impairs exercise capacity in both rats and humans, and increases expression of the mitochondrial uncoupling protein, UCP3, in rodent cardiac and skeletal muscle via activation of the transcription factor, peroxisome proliferator-activated receptor α (PPARα). Unlike long-chain fatty acids however, medium-chain fatty acids do not activate PPARα and do not increase muscle UCP3 expression. We therefore investigated exercise performance and cardiac mitochondrial function in rats fed a chow diet (7.5% kcal from fat), a long-chain triglyceride (LCT) rich diet (46% kcal from LCTs) or a medium-chain triglyceride (MCT) rich diet (46% kcal from MCTs). Rats fed the LCT-rich diet for 15 days ran 55% less far than they did at baseline, whereas rats fed the chow or MCT-rich diets neither improved nor worsened in their exercise capacities. Moreover, consumption of an LCT-rich diet increased cardiac UCP3 expression by 35% and decreased oxidative phosphorylation efficiency, whereas consumption of the MCT-rich diet altered neither UCP3 expression nor oxidative phosphorylation efficiency. Our results suggest that the negative effects of short-term high-fat feeding on exercise performance are predominantly mediated by long-chain rather than medium-chain fatty acids, possibly via PPARα-dependent upregulation of UCP3.

## Background

Metabolic efficiency is a major determinant of endurance performance, determining the speed or power that can be generated for a given level of oxygen consumption [1]. Diet can alter metabolic efficiency, and thereby affect performance, even over a relatively short time. Indeed, we demonstrated that consumption of a high fat diet for 9 days impaired exercise capacity in rats [2] and in similar studies, found that short-term high fat feeding impaired exercise efficiency [3] and cardiac energetics [4] in men, although interestingly the detrimental effects on exercise performance were not seen in highly-trained individuals [5].

We proposed that the mechanism underlying the effects of high fat feeding involve altered mitochondrial function, mediated by changes in transcription of metabolic proteins. Plasma free fatty acid (FFA) levels increase during high fat feeding, and increase fatty acid uptake by cardiac and skeletal muscle [6] activating the nuclear transcription factor, peroxisome proliferator-activated receptor α (PPARα). Expression of fatty acid oxidation enzymes, such as carnitine palmitoyl-transferase 1 (CPT1) and medium-chain acyl-CoA dehydrogenase (MCAD) [7,8] is thereby increased. PPARα activation, however, also upregulates levels of the mitochondrial uncoupling protein UCP3 [9,10]. When activated, UCP3 allows protons to cross the inner mitochondrial membrane independent of ATP synthesis, thereby uncoupling oxidative phosphorylation and decreasing metabolic efficiency (ATP produced/O_2 _consumed). We found that short-term high fat feeding increased cardiac and skeletal muscle UCP3 levels, and uncoupled skeletal muscle mitochondria [2]. Moreover, a similar regime of high-fat feeding in rats increased mitochondrial fatty acid oxidation and uncoupling, resulting in decreasing cardiac efficiency [11].

Activation of PPARα may, however, be influenced by the type of fatty acid available. Long-chain fatty acids act as more potent ligands for PPARα than medium-chain fatty acids, and dietary supplementation of long-chain triglycerides increased muscle UCP3 expression, whereas medium-chain triglyceride feeding did not [12]. As such, medium-chain triglycerides (MCTs), unlike long-chain triglycerides (LCTs), may not impair exercise performance. Moreover, it has been suggested that MCTs might in fact enhance exercise performance [13], because a high MCT load elicits a ketogenic response [14], and ketone bodies improve metabolic efficiency in the isolated, perfused heart [15] and other tissues [16]. There is, however, little conclusive evidence for this [13].

In this study, we investigated the effects of short-term high-fat diets containing either MCTs or LCTs on physical performance and mitochondrial function in rats. Male Wistar rats were fed either standard laboratory chow (7.5% kcal from fat) or one of two high-fat diets containing 46% kcal from either MCTs or LCTs. Endurance capacity was assessed using a motorised treadmill and the rates and efficiency of fatty acid oxidation were measured in isolated cardiac mitochondria, along with levels of UCP3.

## Methods

### Animals and Diets

Male Wistar rats weighing ~250 g (n = 33) were purchased from a commercial breeder (Harlan, Oxfordshire, UK), provided with food and water *ad libitum*, and housed individually to monitor daily food intake under controlled conditions for temperature and humidity on a 12 h:12 h reverse light:dark photoperiod, such that testing was conducted during their active, dark period. All studies were carried out in accordance with UK Home Office and US NIH guidelines, and carried out under UK Home Office personal and project licences. The University of Oxford Animal Ethics Review Committees and the Home Office (London, UK) approved all procedures.

**Figure 1:**
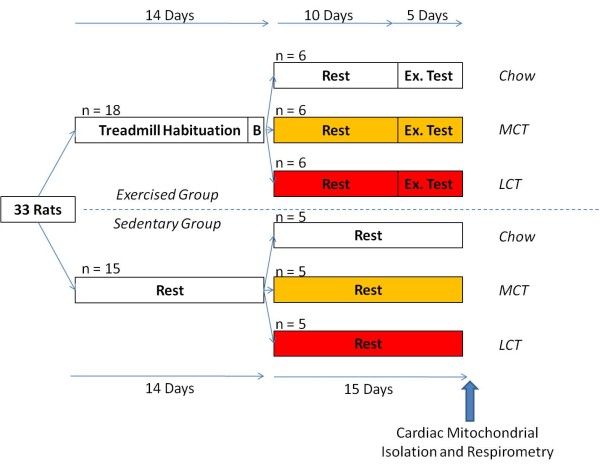
**Timeline of the study showing diet and exercise regimens**. The exercised group is shown on the upper part of the diagram and the sedentary group on the lower part. The colours of boxes indicate the diet consumed with white boxes indicating chow diet, gold boxes indicating MCT rich-diet and red boxes indicating LCT-rich diet. B = Baseline exercise test. Ex. Test = Exercise test.

A timeline of the study is presented in Figure 1. All rats were initially fed a standard laboratory chow (Rat and Mouse No. 1 Maintenance, Special Diet Services, Witham, Essex, UK), which had an Atwater Fuel Energy (AFE) of 3.3 kcal/g, comprising 7.5% from oil, 17.5% from protein and 75% from carbohydrate. All rats continued on this diet as a subgroup (n = 18) was habituated to a motorised treadmill for exercise testing (see below). During the testing period, 11 rats (6 exercised, 5 sedentary) continued to be provided with chow *ad libitum*, whilst 11 rats (6 exercised, 5 sedentary) were switched from standard chow to an LCT-rich diet (Hope Farm, Woerden, Netherlands) and the remaining 11 rats (6 exercised, 5 sedentary) were provided with an MCT-rich diet (Hope Farm, Woerden, Netherlands). The macronutrient compositions and caloric contents of each diet are shown in Table 1.

**Table 1:** Macronutrient components and calorie densities of test diets used in this study.

	Chow	Medium-Chain Triglyceride	Long-Chain Triglyceride
**Energy density (kcal/g)**	3.3	4.6	4.6
*Composition (% of total energy)*			
**Carbohydrate**	75	35	35
**Fat**	7.5	46	46
**Protein**	17.5	19	19
*Fat composition (% of total energy)*			
**C8-C12:0**	0.1	46	-
**C14:0**	0.4	-	0.5
**C14:1**	0.1	-	-
**C16:0**	0.9	-	36.7
**C16:1**	0.2	-	-
**C18:0**	0.1	-	2.8
**C18:1**	2.1	-	5.6
**C18:2**	1.9	-	0.5
**C18:3**	0.2	-	-
**C20-22**	0.4	-	-

### Exercise Capacity

A subgroup of rats (n = 18) were habituated to a motorised treadmill (Columbus Instruments, OH, USA) over a 14 day period, running at gradually increasing belt velocities, whilst the remaining rats (n = 15) remained sedentary. After this period, all exercising rats were proficient at running on the treadmill for 5 min at a velocity of 10 m/min on a 5° incline. From their first encounter with the treadmill, rats were exposed to a small electric stimulus at the rear of the treadmill that delivered a shock of 1.2 mA of current at 3 Hz. An electric fan was used to cool the rats during exercise and circulate air in the treadmill chamber. These rats subsequently underwent baseline testing, comprising three exercise tests on the treadmill on consecutive days, during which the belt velocity was initially set at 10 m/min and increased by 1 m/min^2 ^until the rat fatigued (i.e. could no longer keep pace with the belt) and the distance run recorded.

During the test period, 6 exercising rats continued on the chow diet, 6 exercising rats received the MCT diet and 6 received the LCT diet. Rats were individually housed to monitor calorie intake, and were pair-fed according to food consumption in the chow diet group, such that all rats received the same number of calories during the testing period. Exercising rats were given 10 days to recover from baseline tests and familiarise themselves with the new diets, during which they were re-habituated to the treadmill (5 min at 10 m/min) every other day. After this period, the exercising rats underwent 5 days of performance testing, each comprising three exercise tests separated by > 2 hours.

After the final exercise test on the fifth day, rats were anaesthetised with a 0.8 ml *i.p*. injection of 60 mg.ml^-1 ^sodium pentobarbitone (Sagatal, Rhône Mèrieux, Dublin, Ireland). Following cessation of peripheral nervous function, hearts were excised, rinsed in phosphate-buffered saline (PBS), trimmed to remove extraneous tissue, blotted and weighed. Whole soleus, from one leg of each rat, was trimmed, and heart and soleus snap-frozen using Wollenberger clamps cooled under liquid N_2_.

At the point of heart excision, blood was taken from the thoracic cavity, rapidly centrifuged at 1000 × *g *at 4°C, and the plasma frozen at -80°C for biochemical analysis. Lipoprotein lipase inhibitor (2%) was added to an aliquot of plasma for free fatty acid analysis. Plasma levels of glucose, lactate, β-hydroxybutyrate, triglycerides and total cholesterol, were measured using an ABX Pentra Clinical Chemistry bench-top analyser (Horiba ABX, Montpellier, France). Plasma levels of free fatty acids were measured using a NEFA assay kit (Wako Chemicals GmbH, Neuss, Germany).

### Cardiac Mitochondrial Isolation and Respiratory Measurements

Sedentary rats were fed either the chow (n = 5), MCT (n = 5) or LCT diets (n = 5) for 15 days, pair-fed to the chow group, before being killed as described above. Blood samples were collected, and plasma prepared and analysed as described above. Part of the left ventricle (~100 mg) was snap-frozen for immunoblotting, whilst mitochondria were isolated from the remainder of the hearts of these rats, as described previously [17].

Respiratory rates of rat cardiac mitochondria were measured using a Clark-type oxygen electrode (Strathkelvin Instruments Ltd, Glasgow, UK), as described previously [18]. The chambers were treated identically throughout the experiment, except that one contained 1 μmol GDP added to the respiratory medium at the start of the experiment. GDP is an inhibitor of the mitochondrial uncoupling proteins [19]; therefore differences between the two chambers would indicate the effect of UCP3 on respiration. Oxygen concentrations were recorded in the absence of mitochondria, and after addition of approximately 0.5 mg mitochondrial protein. Malate (5 mM) plus palmitoyl-carnitine (0.04 mM), were added as substrates, before state III respiration was stimulated by the addition of 100 nmoles of ADP. State IV respiration, which occurred when all the ADP added to the respiration medium had been phosphorylated, was subsequently measured. ADP/O ratios (the ratios of ADP molecules phosphorylated for each oxygen atom consumed) were calculated as described by Estabrook [20]. ADP/O ratios are independent of the rates of oxygen consumption and give a more precise measure of oxidative phosphorylation efficiency than can be inferred from respiration rates alone.

### Immunoblotting

Levels of UCP3 were measured in cardiac and skeletal muscle lysates by immunoblotting, as described previously [17], using a polyclonal rabbit anti-UCP3 antibody (Millipore, Billerica, MA, USA) at a concentration of 1:1000 in 5% milk TBS-Tween. The secondary antibody used was anti-rabbit IgG peroxidase conjugate polyclonal antibody (Autogen Bioclear, Wiltshire, UK) used at a concentration of 1:3500 in 5% milk TBS-Tween.

### Statistical Analysis

Results are expressed as means ± SEM. All data were checked for normal distribution. Analysis of variance (ANOVA) with repeated measures and Bonferroni *post-hoc *independent unpaired t-tests were used to determine differences between diet groups. Paired t-tests were used to determine within-group differences between baseline measurements and the test period, and isolated mitochondrial measures +/- GDP from the same animal. Data were considered statistically significant at p < 0.05.

## Results

### Food intakes and body weights

All 33 rats began the study with approximately the same body weight 250 ± 3 g. Sedentary LCT-fed rats gained more weight over 15 days of feeding than either the sedentary chow-fed or MCT-fed rats, finishing the study with 20% greater body weights than sedentary chow-fed rats (p < 0.01) (Table 2). Heart weights, and heart:body weights were the same in all sedentary rats at the end of the study. Sedentary LCT-fed rats had 59% greater epididymal fat pad weights than sedentary chow-fed animals (p < 0.05), reflecting greater whole-body adiposity, however epididymal fat pad weights in the sedentary chow-fed and MCT-fed rats were the same (Table 2).

**Table 2:** Daily dietary intake, body weights, growth rates, heart weights and blood plasma metabolites from sedentary and exercised rats fed chow, MCT and LCT diets.

	Sedentary Rats			Exercised Rats		
	**Chow**	**MCT**	**LCT**	**Chow**	**MCT**	**LCT**
	(n = 6)	(n = 6)	(n = 6)	(n = 5)	(n = 5)	(n = 5)
*Morphological data*						
**Body weight (g)**	322 ± 3	327 ± 12	365 ± 16**	287 ± 15	276 ± 8	262 ± 8
**Heart weight (g)**	0.81 ± 0.02	0.88 ± 0.03	0.91 ± 0.05	0.77 ± 0.05	0.77 ± 0.01	0.74 ± 0.01
**HW/BW × 1000**	2.6 ± 0.1	2.7 ± 0.1	2.5 ± 0.1	2.7 ± 0.4	2.7 ± 0.3	2.8 ± 0.2
**Epididymal fat weight (g)**	2.7 ± 0.4	3.2 ± 0.3	4.3 ± 0.6*	3.3 ± 0.4	3.9 ± 0.4	2.8 ± 0.2
*Non-fasting plasma metabolites*						
**Glucose (mM)**	13.7 ± 2.6	14.7 ± 1.6	14.2 ± 1.4	7.2 ± 1.7	9.7 ± 1.5	8.5 ± 1.0
**Lactate (mM)**	4.8 ± 2.3	4.6 ± 1.3	4.7 ± 0.7	2.7 ± 1.1	3.5 ± 0.5	3.4 ± 0.7
**Free fatty acids (mM)**	0.22 ± 0.06	0.31 ± 0.03	0.23 ± 0.02	0.51 ± 0.12	0.47 ± 0.12	0.32 ± 0.09
**Triglycerides (mM)**	1.1 ± 0.2	2.3 ± 0.3*	2.3 ± 0.4*	0.6 ± 0.1	0.85 ± 0.2	0.99 ± 0.3
**Cholesterol (mM)**	1.3 ± 0.1	1.4 ± 0.2	1.9 ± 0.1*^,‡^	1.5 ± 0.1	1.3 ± 0.1*	1.8 ± 0.1*^,‡‡^
**β-Hydroxybutyrate (mM)**	0.41 ± 0.05	0.46 ± 0.07	0.30 ± 0.03	0.21 ± 0.02	0.29 ± 0.06	0.20 ± 0.07

(HW/BW = heart weight/body weight)

* p < 0.05, ** p < 0.01 vs. chow-fed rats of same experimental test group, i.e. sedentary vs. sedentary, exercised vs. exercised.

^‡ ^p < 0.05, ^‡‡ ^p < 0.01 vs. MCT-fed rats of same experimental test group, i.e. sedentary vs. sedentary, exercised vs. exercised.

(HW/BW = heart weight/body weight).

There was no difference in end body weights between any of the exercised rats, although both exercising MCT and LCT-fed rats had lower body weights (p < 0.01 and p < 0.001, respectively) than their sedentary counterparts. Heart:body weights were the same for all exercising rats, and were the same as their sedentary counterparts. There were no differences in epididymal fat pad weights between any of the exercising groups (Table 2).

### Non-fasting plasma metabolites

There were no significant differences in plasma glucose levels between any of the dietary groups in either sedentary or exercising rats, however, exercising MCT-fed and LCT-fed rats had significantly lower glucose levels than their sedentary counterparts (p < 0.05 and p < 0.01, respectively). Plasma lactate and free fatty acid levels were not altered by diet or exercise. Plasma triglyceride levels were higher in both sedentary MCT and LCT-fed rats than in sedentary chow-fed rats (p < 0.05), but lower in exercised rats than their chow-fed counterparts (p < 0.05 for chow and LCT-fed rats, p < 0.01 for MCT-fed rats). Plasma cholesterol levels were greater in LCT-fed rats than in chow-fed or MCT-fed rats (p < 0.05). There were no significant differences in plasma cholesterol levels between exercised and sedentary rats within any diet group. There was no significant difference in plasma β-hydroxybutyrate levels between any diet group in either the sedentary or exercised rats (Table 2).

### Exercise capacities

**Figure 2:**
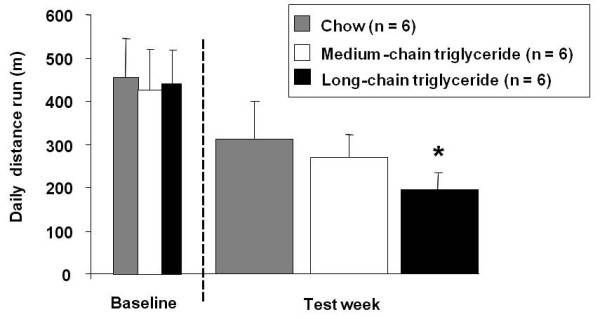
**Effect of dietary fat on endurance capacity**. Average distances run by rats at baseline and during running days 1-5, whilst eating chow (grey), MCT-rich (white) or LCT-rich (black) diets. * p < 0.05 compared with baseline.

All rats ran similar distances on the treadmill during their baseline tests, running an average of 437 ± 78 m/day (Figure 2). During the five test days, rats fed the MCT and chow diets showed no significant change in performance compared with their baseline performances, whereas rats fed the LCT diet showed a significant decline in treadmill running performance (Figure 2). Over the week, rats fed the LCT diet ran 55% less far on average than they did at baseline (p < 0.05) (194 ± 41 m vs. 429 ± 79 m), whereas the distance run by chow-fed rats (312 ± 89 m vs. 455 ± 77 m) and MCT-fed rats (270 ± 53 m vs. 427 ± 80 m) was not significantly different to that at baseline (Figure 2).

### Cardiac mitochondrial respiration

**Figure 3:**
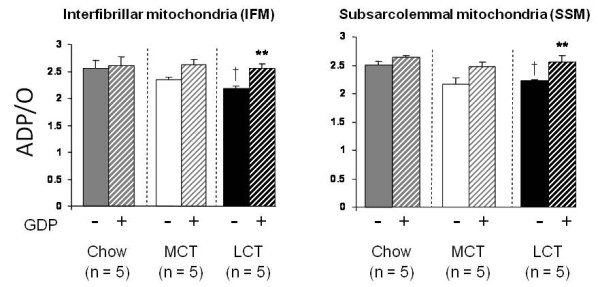
**Effect of dietary fat on cardiac mitochondrial respiration in sedentary rats**. ADP/O ratios of mitochondria isolated from the hearts of chow (grey), MCT-rich (white) or LCT-rich (black) diet fed rats in the presence and absence of GDP (UCP3 inhibitor), with palmitoyl-L-carnitine plus malate as substrate. ** p < 0.01 compared with same mitochondria in the absence of GDP. ^† ^p < 0.05 compared with chow-fed mitochondria in the absence of GDP.

Maximal ADP-stimulated respiration rates (State III) and ADP-independent respiration rates (State IV) were similar across all three dietary groups in both interfibrillar and subsarcolemmal mitochondria from rat heart. Addition of GDP decreased State III respiration rate in subsarcolemmal mitochondria from the LCT-fed group by 37% (p < 0.05), but did not significantly alter respiration rates in any other mitochondria (Table 3). In both interfibrillar and subsarcolemmal mitochondria from LCT-fed rats, ADP/O was significantly lower than in the corresponding mitochondria from the chow-fed group (p < 0.05), and addition of GDP increased the ADP/O ratio (p < 0.05) such that there was no difference between the groups (Figure 3).

**Table 3:** State III (ADP-stimulated) and state IV (ADP phosphorylated) respiration rates of interfibrillar and subsarcolemmal cardiac mitochondria from rats fed chow, medium-chain triglyceride or long-chain triglyceride rich diets, respiring with palmitoyl-carnitine in the presence and absence of GDP.

	Interfibrillar Mitochondria		Subsarcolemmal Mitochondria	
	- GDP	+ GDP	- GDP	+ GDP
***State III***				
**Chow**	113 ± 18	109 ± 7	105 ± 14	90 ± 18
**MCT**	98 ± 19	95 ± 20	93 ± 13	87 ± 6
**LCT**	121 ± 27	104 ± 33	103 ± 21	65 ± 13*
***State IV***				
**Chow**	37 ± 4	34 ± 3	40 ± 3	37 ± 3
**MCT**	38 ± 6	35 ± 7	31 ± 5	28 ± 4
**LCT**	40 ± 9	37 ± 8	47 ± 13	41 ± 6

n = 5 per group.

* p < 0.05 compared with same mitochondria in the absence of GDP.

### Uncoupling protein 3 levels

**Figure 4:**
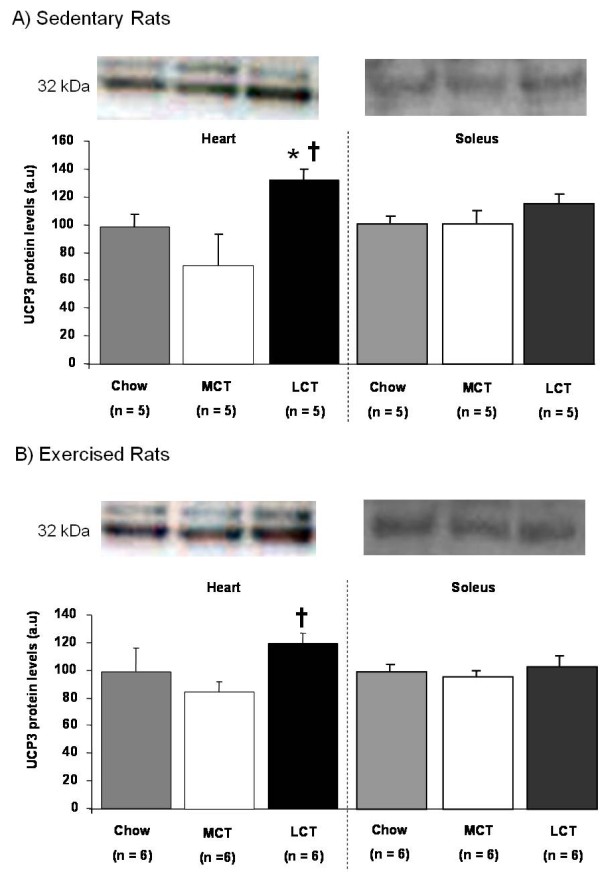
**Effects of high-fat feeding on cardiac and skeletal muscle uncoupling protein 3 (UCP3) levels in A) sedentary and B) exercised rats fed chow (grey), MCT-rich (white) or LCT-rich (black) diets, with sample blots shown**. * p < 0.05 compared with chow-fed rats. ^† ^p < 0.05 compared with MCT-fed rats.

In sedentary rats, cardiac UCP3 levels were 35% higher in LCT-fed rats than chow-fed rats (p < 0.05) and 93% higher than in MCT-fed rats (p < 0.05) (Figure 4A). In exercising rats, cardiac UCP3 levels were 37% higher in LCT-fed rats than in MCT-fed rats (p < 0.05) (Figure 4B). No differences were seen in skeletal muscle UCP3 levels from rats fed the different diets, whether sedentary or exercised (Figure 4).

## Discussion

In heart and skeletal muscle, metabolic gene expression and thus mitochondrial function and efficiency (ATP produced/O_2 _consumed) can be influenced by dietary composition [21,22]. In particular, high fat feeding activates the transcription factor, PPARα, promoting the expression of genes associated with relatively inefficient fatty acid oxidation including the mitochondrial uncoupling proteins [21,22]. Metabolic efficiency is a key determinant of endurance performance [1], and we have shown previously that short-term consumption of a high-fat diet impaired exercise capacity in rats [2] and decreased exercise efficiency in humans [3]. Unlike long-chain fatty acids however, medium-chain fatty acids do not activate PPARα [12], and therefore in this study we investigated the effects of short-term high-fat feeding on exercise capacity and cardiac mitochondrial function using diets rich in LCTs and MCTs.

In sedentary rats, LCT-feeding increased body weights and adiposity compared with chow-fed or MCT-fed rats, though not in exercising rats, and heart weight to body weight ratios were the same for all animals. As expected, both MCT and LCT diets increased circulating triglyceride levels, though only in sedentary animals, and LCT-feeding increased cholesterol in both sedentary and exercising rats. In agreement with other studies, consumption of a LCT-rich diet increased cardiac UCP3 expression in sedentary rats, whereas the MCT-rich diet did not [12], and here we have extended this finding to exercised rats. Moreover, whilst no diet altered ADP-stimulated fatty acid oxidation rates or State IV respiration rates in cardiac mitochondria, the mitochondria from LCT-fed rats had a lower oxidative phosphorylation efficiency than those from either chow-fed or MCT-fed rats as shown by a decreased ADP/O ratio, and this effect was ameliorated by the UCP3 inhibitor, GDP. ADP/O ratios are calculated independently of respiration rates, and give a more sensitive measure of the coupling of fuel oxidation and ADP phosphorylation than can be inferred from respiration rates alone [20]. Unfortunately, due to the time commitment of carrying out exercise tests on 18 rats, we were unable to isolate mitochondria from the hearts of exercised rats and instead used a sedentary group. This additional data would have been interesting, and whilst the effects of diet on UCP3 levels were similar in the two groups, it cannot be excluded that respiration might have been different in the exercised and sedentary rats.

Curiously, unlike our findings in the heart we did not find that consumption of the LCT-rich diet increased UCP3 levels in soleus. This is perhaps in contrast with our finding that a 55% fat diet increased UCP3 in soleus [2], although it should be noted that the diets used in this study had a lower total fat content than used previously and a higher carbohydrate content. It should also be noted that as, with our previous study using a 55% fat diet [2], we measured plasma metabolite levels in the fed state, and although we saw slightly higher plasma FFA levels in sedentary rats fed the MCT and LCT diets compared with their chow-fed counterparts this did not reach significance. Differences in plasma FFA levels between animals fed the two diets would likely be more apparent in the fasted state and could underlie the increased cardiac UCP3 levels in the MCT and LCT-fed rats via PPARα-activation [9], whilst not being sufficiently elevated to elicit activation in skeletal muscle, which has a lower fatty acid oxidation capacity than heart.

The lack of an effect of the MCT and LCT diets on skeletal muscle UCP3, compared with our previous high fat diet [2], may also explain another discrepancy. Previously, we found that the combination of a high fat diet and sustained exercise stress resulted in a 20% increase in heart/body weights [2]. We speculated that this may have represented an adaptive response to increase cardiac output and thereby enhance oxygen delivery to muscles in which there was notable uncoupling. We did not find an increase in heart/body weights here in rats fed either MCT or LCT diets, and perhaps this is in agreement with unchanged skeletal muscle UCP3 levels, although again we did not measure mitochondrial function directly in skeletal muscle mitochondria in this investigation.

An intensive exercise challenge over 5 days resulted in the exercised rats having lower body weights than their sedentary counterparts, along with decreased blood glucose, lactate, β-hydroxybutyrate and triglyceride levels, but increased free fatty acid levels, perhaps resulting from exercise-induced lipolysis. Over the five days of treadmill running, the exercise performance of chow-fed and MCT-fed rats was maintained at baseline levels, whereas LCT-fed rats were unable to sustain their performance level, running on average 55% less far than they did at baseline. This decrement is remarkably similar to the degree to which performance fell in high-fat fed rats in our previous study [2], despite a somewhat lower dietary fat content in this study (47% compared with 55% previously). Taken together with the elevated cardiac UCP3 levels in these rats and altered mitochondrial function in the sedentary animals, this might suggest that the detrimental effect of short-term high fat feeding resulted from long chain fatty acid activation of PPARα, and consequent loss of metabolic efficiency.

Notably, whilst the MCT-fed rats did not exhibit a significant loss of exercise capacity over five days of treadmill running, neither did they improve, either compared with their own baseline or compared to chow-fed rats. It has been theorised that MCT-rich diets might improve energy utilisation during exercise[13], perhaps through increased ketogenesis [14], however there is little conclusive evidence for this. One study reported an increased swimming endurance capacity in mice fed an MCT-rich diet compared with LCT-fed mice over 6 weeks, yet in this study both groups of mice were initially untrained at the task and both improved throughout the trial [23]. As MCT-fed mice improved swim capacity more than LCT-fed counterparts [23], this study, like ours, in fact demonstrated a detrimental effect of LCT-feeding compared with MCT, rather than a compelling beneficial effect of MCTs on performance. Interestingly, MCT-feeding did not significantly increase plasma ketone body levels in mice in this study [23], nor in sedentary or exercised rats in our study, perhaps because carbohydrate levels in the diets used were not particularly low.

In clinical trials, there is also little support for a training benefit from MCT-rich diets. One study demonstrated no difference in cycling performance amongst subjects fed 400 kcal as MCTs, LCTs or carbohydrates, despite increased ketogenesis in MCT-fed subjects [24]. Meanwhile others have reported a drop in cycling performance when dietary carbohydrates are replaced by MCTs [25,26], although in one case this was possibly due to gastrointestinal upset [25].

Finally, it is likely that the upregulation of UCP3 in response to excess dietary fat forms part of an adaptive response. There has been some controversy concerning the physiological function of UCP3 with many possible roles suggested. There is compelling evidence to support a mild uncoupling function [27-29] of UCP3. This uncoupling probably plays a role in lowering reactive oxygen species (ROS) production [30], with both the mitochondrial membrane potential and ROS generation increased in UCP3^-/- ^mice and following inhibition of UCP3 with GDP [30]. Alternatively, it has been suggested that UCP3 is involved in the export of fatty acid anions or lipid peroxides from the mitochondrial matrix [31]. Symmetry analysis of the structure of UCP3, alongside that of mitochondrial carriers known to transport fatty acid derivatives, however, does not support this hypothesis, with the UCPs lacking hydrophobic residues in their binding pocket on the matrix side [32]. The authors of this study conclude that the substrate of the UCPs is more likely to be small carboxylic or keto acids, transported in symport with protons [32]. None of these alternative functions however, exclude a mild uncoupling effect, whereby protons re-enter the matrix independently of ATP-synthesis, decreasing the efficiency of oxidative phosphorylation (ATP synthesised per O_2 _consumed).

## Conclusions

In conclusion, our study demonstrates that consumption of an LCT-rich diet impaired exercise capacity in rats over just 15 days of feeding. The LCT diet also increased cardiac UCP3 levels, which decreased efficiency of oxidative phosphorylation. Cardiac mitochondrial uncoupling may underlie the decrement in performance that we report here, although curiously we did not observe any changes in skeletal muscle UCP3 levels. Consumption of an MCT-rich diet did not alter cardiac UCP3 expression or oxidative phosphorylation efficiency at the mitochondria, and neither worsened nor improved exercise performance, compared with a chow diet. Whilst there remains no compelling case for an ergonomic benefit from MCT-rich diets, there is a strong case against the consumption of LCT-rich diets during endurance training.

## Abbreviations

AFE: Atwater Fuel Energy; CPT-1: Carnitine Palmitoyltransferase-1; FFA: Free Fatty Acid; LCT: Long Chain Triglyceride; MCAD: Medium Chain Acyl-CoA Dehydrogenase; MCT: Medium Chain Triglyceride; PPARα: Peroxisome Proliferator-Activated Receptor α; UCP3: Uncoupling Protein 3

## Competing interests

The authors declare that they have no competing interests.

## Authors' contributions

AJM designed the studies, carried out exercise testing, mitochondrial respirometry and western blotting and co-wrote the manuscript. NSK, SEL, LEC and MC out exercise testing, mitochondrial respirometry and western blotting. KC contributed to study design and co-wrote the manuscript. All authors read and approved the final manuscript.
